# Supplementary material for: Transcriptomics Using the Enriched Arabidopsis Shoot Apex Reveals Developmental Priming Genes Involved in Plastic Plant Growth under Salt Stress Conditions
Source: Plants (Basel). 2022 Sep 28;11(19):2546. doi: 10.3390/plants11192546 (PMC9570865; doi:10.3390/plants11192546)
Supplement: Supplementary file 1 [file plants-11-02546-s001.zip › Revised Supplementary Material.pdf]

Supplemental Figures

Transcriptomics using the enriched Arabidopsis shoot apex reveals developmental priming genes involved in plastic plant growth under salt stress conditions

Ok-Kyoung Cha, Soeun Yang, and Horim Lee*

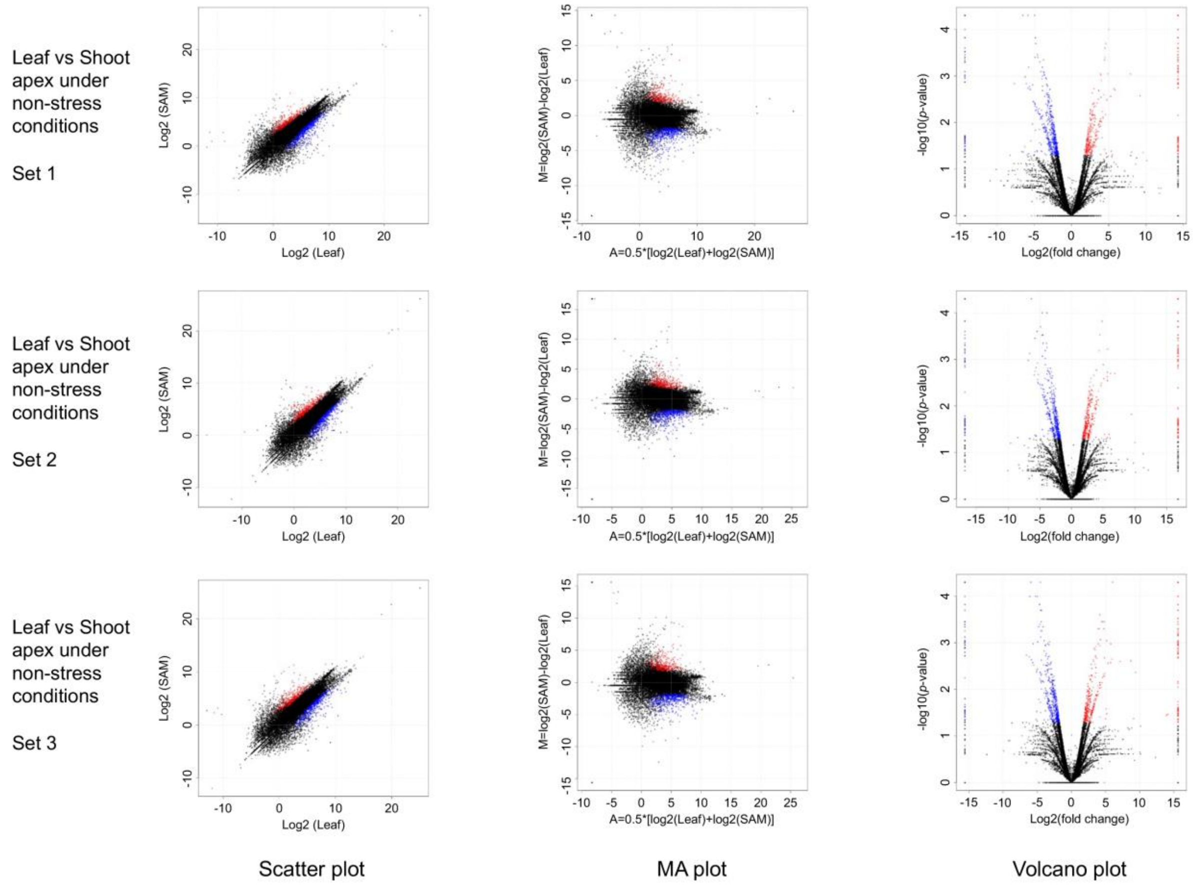

Figure S1. Quality validation of RNA-seq experiments using leaf and shoot apex tissues under control conditions. Differentially expressed genes (DEGs) were validated via Scatter, MA and Volcano plots. **Scatter plot**: after setting the X-axis and Y-axis as the log2 value of the gene expression level for each group, the gene is highlighted for the applied filter value. **MA plot**: The X-axis is the value obtained by multiplying the log2 sum of gene expression levels in each group by 0.5, and the Y-axis is the log2 difference value of the gene expression levels of each group. After setting the X- and Y-axis, the gene is highlighted for the applied filter value. **Volcano plot**: after setting the X-axis to log2(fold change) and the Y-axis to -log10(*p*-value), the gene is highlighted for the applied filter value.

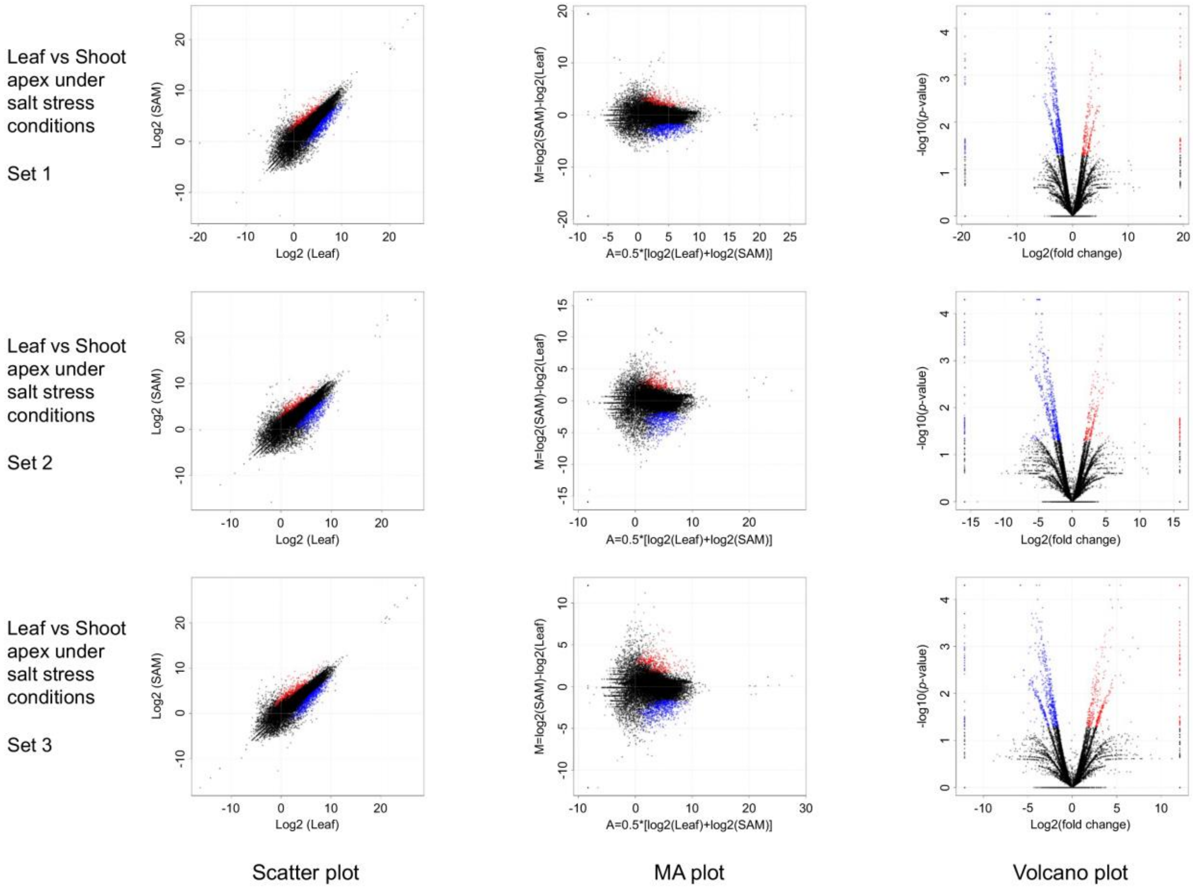

Figure S2. Quality validation of RNA-seq experiments using leaf and shoot apex tissues under salt stress conditions. Differentially expressed genes (DEGs) were validated via Scatter, MA and Volcano plots.

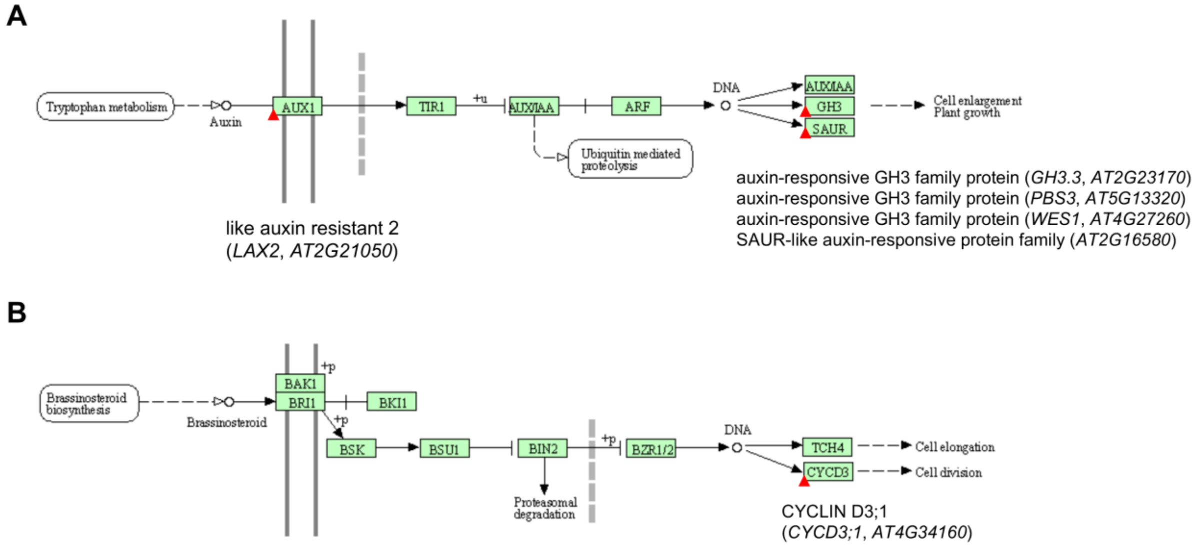

Figure S3. Upregulated developmental priming genes in a KEGG pathway. Seventy DEGs as an upregulated developmental priming genes displaying upregulation in the shoot apex compared to leaf under both control and salt stress conditions were applied to the DAVID program for analyzing a KEGG pathway. Interestingly, only one KEGG term (ath04075:Plant hormone signal transduction) was revealed with 7.314-fold enrichment (p=0.002474). Applied genes in KEGG pathway analysis were indicated as red arrow heads in auxin (**A**) and brassinosteroid (**B**) pathways involved in cell elongation and division.

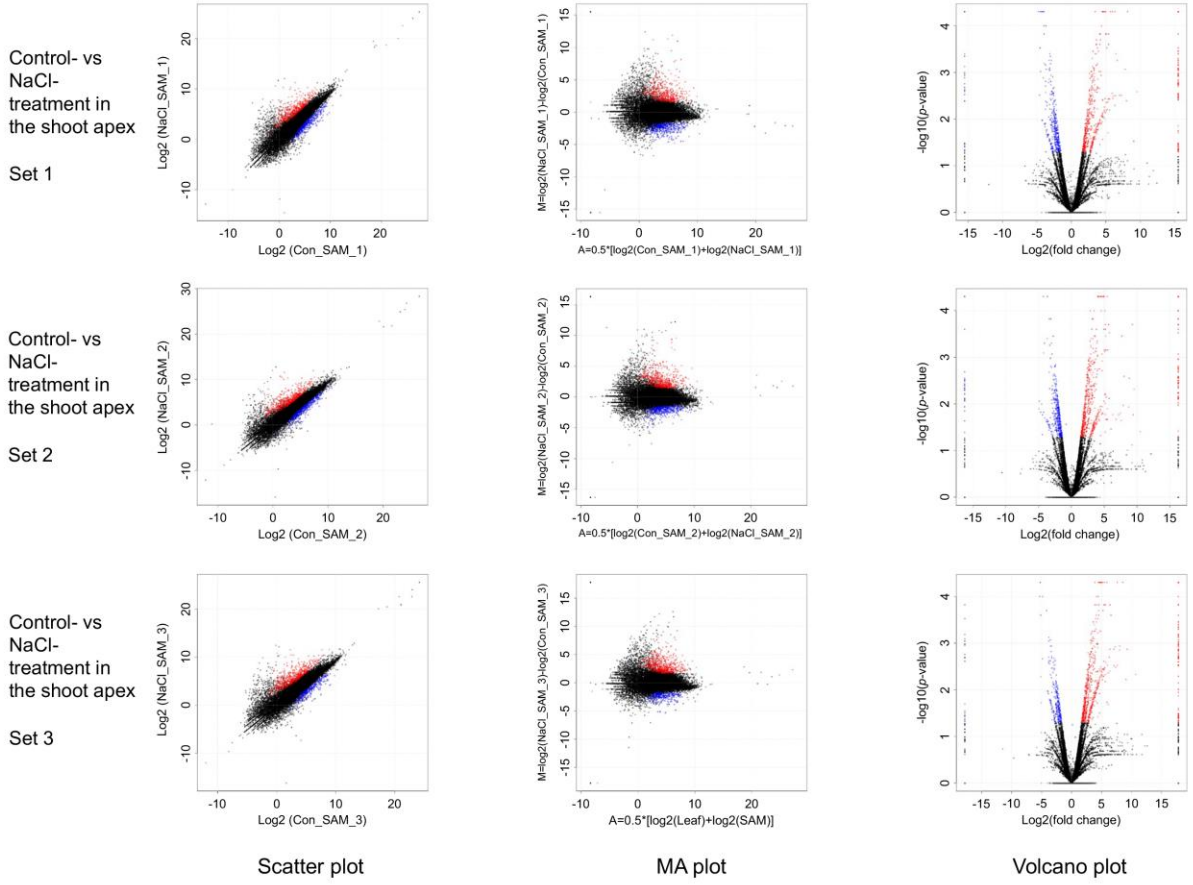

Figure S4. Quality validation of RNA-seq experiments using shoot apex tissues under control or NaCl conditions. Differentially expressed genes (DEGs) were validated via Scatter, MA and Volcano plots.

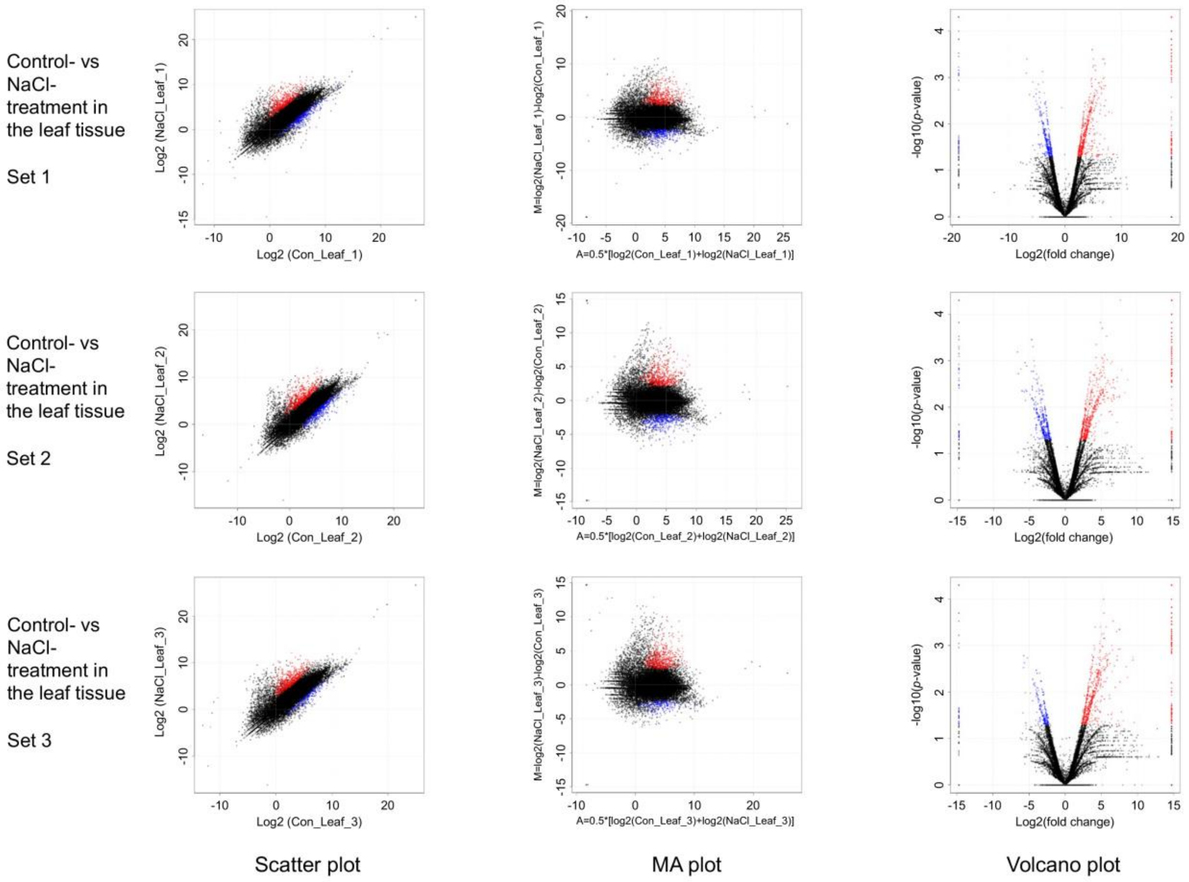

Figure S5. Quality validation of RNA-seq experiments using leaf tissues under control or NaCl conditions. Differentially expressed genes (DEGs) were validated via Scatter, MA and Volcano plots.

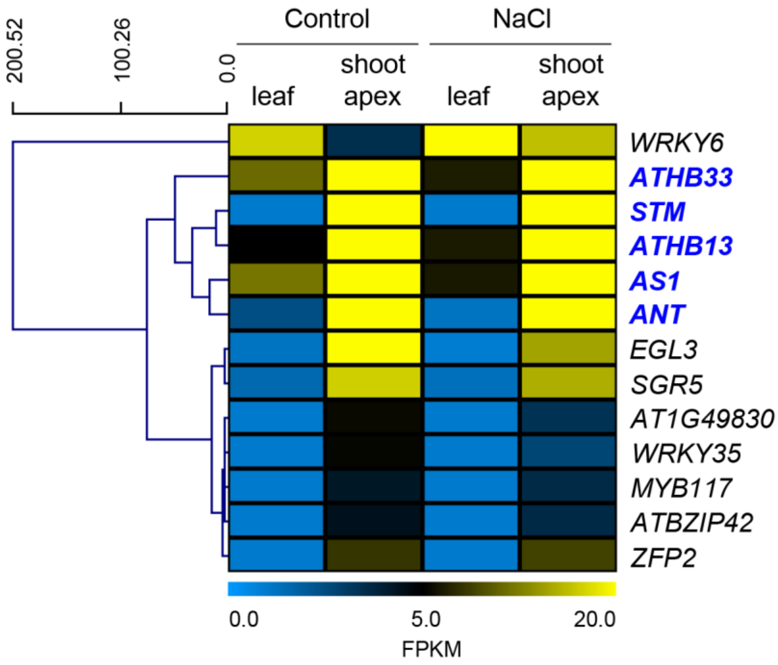

Figure S6. Clustering analysis of TF genes classified as developmental priming genes. Heatmap is the result of hierarchical clustering via MeV 4.9.0 with the current metric Euclidean Distance. The FPKM value of each gene was used with the average value of three replicates.
